# GLP-1 receptor agonist ameliorates obesity-induced chronic kidney injury via restoring renal metabolism homeostasis

**DOI:** 10.1371/journal.pone.0193473

**Published:** 2018-03-28

**Authors:** Chengshi Wang, Ling Li, Shuyun Liu, Guangneng Liao, Lan Li, Younan Chen, Jingqiu Cheng, Yanrong Lu, Jingping Liu

**Affiliations:** 1 Key Laboratory of Transplant Engineering and Immunology, West China Hospital, Sichuan University, Chengdu, China; 2 Division of Nephrology, Kidney Research Institute, West China Hospital, Sichuan University, Chengdu, China; University Medical Center Utrecht, NETHERLANDS

## Abstract

Increasing evidence indicates that obesity is highly associated with chronic kidney disease (CKD). GLP-1 receptor (GLP-1R) agonist has shown benefits on kidney diseases, but its direct role on kidney metabolism in obesity is still not clear. This study aims to investigate the protection and metabolic modulation role of liraglutide (Lira) on kidney of obesity. Rats were induced obese by high-fat diet (HFD), and renal function and metabolism changes were evaluated by metabolomic, biological and histological methods. HFD rats exhibited systemic metabolic disorders such as obesity, hyperlipidemia and impaired glucose tolerance, as well as renal histological and function damages, while Lira significantly ameliorated these adverse effects in HFD rats. Metabolomic data showed that Lira directly reduced renal lipids including fatty acid residues, cholesterol, phospholipids and triglycerides, and improved mitochondria metabolites such as succinate, citrate, taurine, fumarate and nicotinamide adenine dinucleotide (NAD^+^) in the kidney of HFD rats. Furthermore, we revealed that Lira inhibited renal lipid accumulation by coordinating lipogenic and lipolytic signals, and partly rescued renal mitochondria function via Sirt1/AMPK/PGC1α pathways in HFD rats. This study suggested that Lira alleviated HFD-induced kidney injury at least partly via directly restoring renal metabolism, thus GLP-1R agonist is a promising therapy for obesity-associated CKD.

## Introduction

Chronic kidney disease (CKD), characterized by progressive destruction of renal mass and slow loss of renal function, is the leading cause to end-stage kidney disease (ESKD) and renal failure[1].Obesity is an epidemic disease and contributes to type 2 diabetes and cardiovascular diseases with high mortality[2]. However, increasing data indicate that obesity is also highly linked with the onset and progression of CKD[3–5]. Epidemiologic studies reported that obesity was associated with a 70% increased risk of microalbuminuria compared to lean subjects[6].High body mass index (BMI) has been considered as one of the strongest risk factors for new-onset CKD[3], and higher baseline BMI remained an independent predictor for ESKD after adjustments for blood pressure (BP) and diabetes mellitus (DM) [7].Obesity may initiate the slow development of kidney dysfunction and lesions including mesangial expansion, glomerular hypertrophy and glomerulosclerosis[8]. The mechanism of obesity-induced kidney injury is complex and multiple factors such as oxidation stress, inflammation, transforming growth factor-β (TGF-β), lipotoxicity, renin-angiotensin system and adipocyte-derived hormones are involved [8, 9]. As the increased prevalence of obesity over the world, it is of great clinical benefits to find therapies for obesity-associated CKD.

Glucose lowering drugs such as Glucagon-like peptide-1 (GLP-1) receptor agonists and sodium-dependent glucose transporters 2 (SGLT2) inhibitors have shown potential protective roles in kidney diseases, and SGLT2 blockers could affect glomerular filtration rate (GFR) and protein excretion in diabetic nephropathy (DN), while GLP-1R agonists reduced urinary albumin excretion in DN [10–12]. GLP-1 is a polypeptide hormone secreted from intestines, which can regulate blood glucose level in diabetic patients, and promote pancreatic cells proliferation in DM models [13, 14]. GLP-1 exerts its biological actions via binding to its receptor (GLP-1R) on the surface of various cells. Besides to pancreatic β-cells,GLP-1R has also been found in kidney cells such as glomerular endothelium and mesangial cells [11, 15].Previous studies have proved the benefits of GLP-1R agonist therapy on acute and chronic kidney diseases[10–12]. GLP-1R agonist exendin-4 (Ex-4) ameliorated cisplatin-induced acute renal tubular cell injury and apoptosis, while inhibition of GLP-1Rabolisheditsrenoprotectiveeffect [10].GLP-1R agonist lira glutide (Lira) alleviated DN in mice through inhibiting glomerular superoxide and NADPH oxidase[16]. Recent study further reported that GLP-1R agonist Ex-4 inhibited renal cholesterol accumulation and inflammation in diabetic apoE knockout mice[17].However, the direct role of GLP-1R agonist on renal metabolic abnormalities in obesity remains not clear.

In this study, we aim to evaluate the renal protection effect of Lira in the obese rats induced by HFD. By using metabolomic, biological and histological methods, we further revealed the metabolic modulation role of GLP-1 on obesity-induced kidney injury.

## Materials and methods

### Ethical statement

All animal work were conducted under the approved guidelines of Sichuan University (Chengdu, China) and approved by the Animal Care and Treatment Committee of Sichuan University (Chengdu, China). The methods were performed in accordance with approved guidelines.

### Animal experiment

Male Sprague-Dawley (SD) rats were purchased from Experimental Animal Center of Sichuan University (Chengdu, China). Animals were housed in pairs of two in cages with controlled temperature(20–22°C), humidity (40–60%), and 12 h cycles of light and darkness, and fed with standard chow and tap water ad libitum during one-week acclimation period. Then all rats were randomly divided into control (n = 10), HFD (n = 10) and HFD +Lira (n = 10) group. Control rats were fed with regular diet (10% fat, 20% protein, and 70% carbohydrate, Chengdu Dashuo Biological Technology Co., Ltd., Chengdu, China), and HFD rats were fed with high-fat diet (63.4% regular diet plus 20% lard, 15% sucrose, 1.5% cholesterol, 0.1% sodium cholate). At 12 weeks after HFD, rats in HFD + Lira group were twice-daily subcutaneously injected with Lira (0.1 mg/kg, Novo Nordisk, Denmark) for 12further weeks, and rats in HFD group were given PBS. The health condition of animals was monitored every day, and none of them showed severe signs of illness or death due to the experimental treatment. To relieve suffering, rats were sacrificed under deeply anesthetized by intraperitoneal administration of pentobarbital sodium overdose at 24 weeks after HFD experiment beginning. The samples of blood, urine and kidney from rats were collected, respectively.

### Biochemical measurement

Clinical biochemistry analysis was performed on an Automatic Biochemistry Analyzer (Cobas Integra 400 plus, Roche) by commercial kits with the following parameters: blood glucose (BG), cholesterol (TC), triglyceride (TG),low-density lipoprotein cholesterol (LDL-C), high-density lipoprotein cholesterol (HDL-C), blood urea nitrogen (BUN), creatinine (CREA), Aspartate transaminase (AST), Alanine transaminase(ALT),creatine kinase(CK) and urinary albumin to creatinine ratio (UACR). The creatinine clearance (CC) was calculated as previously described[18].

### Oil Red O (ORO) staining

Fresh renal tissues obtained from rats were made to 5 μm frozen sections, and then incubated with Oil Red O solution (KeyGen Biotech Co. Ltd., Nanjing, China) at room temperature for 30 min. After washing twice with PBS, the stained sections were observed on a light microscope (Carl Zeiss, Germany).

### Measurement of lipid peroxidation

Renal lipid peroxidation was measured by malondialdehyde (MDA, Beyotime Institute of Biotechnology, China) kit according to the manufacturer’s instructions. Briefly, the supernatant of renal homogenate was added to TBA solution and incubated at 100 °C for 15 min, and then placed on ice to terminate the reaction. After centrifugation, the supernatant of reaction solution was measured by microplate reader (BioTek, MQX200, USA) at 535 nm.

### 1H NMR analysis of kidney tissue

Renal tissues were extracted by chloroform/methanol method as previously described. The aqueous extracts were lyophilized and dissolved in 500 μl PBS containing 50% (v/v) D_2_O and 0.001% (w/v) 3-trimethylsilyl propionic acid-d4 sodium salt (TSP, reference standard, Cambridge Isotope Laboratories, USA). The lyophilized lipid extracts were dissolved in 500 μl deuterated chloroform (CDCl_3_) containing 0.03% (w/v) tetramethylsilane (TMS, reference standard, Cambridge Isotope Laboratories). Nuclear Magnetic Resonance Spectroscopy (NMR) analysis was performed on a Bruker Avance II 600-MHz spectrometer (Bruker Biospin, Germany) at 298 K with a 5-mm PATXI probe. The ^1^H NMR spectra of renal extracts was acquired by a NOESY-presaturation (NOESYGPPR1D) pulse sequence (RD-90°-t1-90°-tm-90°-acq) with following parameters: relaxation delay (RD) = 5 s, mixing time = 100 ms, acquisition time = 2.48 s. The 90° pulse length was about 10 μs, and 64 scans were collected into 32 K data points with a spectral width of 11 ppm. The free induction decay(FIDs)were weighted by an exponential function with a 0.3 Hz line broadening and zero-filled to 32 k data points prior to fourier transform (FT). The chemical shifts were referenced to TSP or TMS at 0 ppm.

### Multivariate data analysis

NMR spectra were manually phased and baseline corrected, and then binned into equal widths (0.003 ppm) corresponding to the 0.5–9.5 ppm (aqueous extract) and 0.5–5.5 ppm (lipid extract) regions by MestReC software (version 4.9.9.9, Mestrelab Research, Spain). The integrals were normalized to the sum intensity of each spectrum excluding the water (4.7–5.2 ppm) regions. The normalized bins were then subjected to principal component analysis (PCA) and orthogonal projection to latent structure with discriminant analysis (OPLS-DA) by SIMCA-P (version 11.5, Umetrics, Sweden). OPLS-DA models were validated by a seven-fold cross validation method with a permutation test to avoid over-fitting. Statistical total correlation spectroscopy (STOCSY) analysis of metabolites was performed by MATLAB R2009a software (Mathworks, Natick, MA), and the OPLS-DA coefficient plot was generated with an in-house developed MATLAB script as previously described [19].

### Mitochondrial ROS (mtROS) assay

The kidney tissue mtROS was measured by mitochondrial superoxide indicator (MitoSOX, Molecular Probes, Thermo Fisher Scientific, USA) according to the manufacturer’s instructions. Briefly, fresh renal tissues were made to 5 μm frozen sections, and then incubated with MitoSOX solution (2 μM) at 37°C for 10 min. After washing with PBS, the stained sections were observed on a fluorescent microscope (Axioplan2, Carl Zeiss, Germany).

### Measurement of kidney ATP

Renal tissue adenosine triphosphate (ATP)was measured by Luciferase ATP Assay Kit (Beyotime, China) according to the manufacturer’s instructions. Briefly, 20 mg tissues were lysed with 200 μl lysis buffer on ice bath, and then centrifuged 12000g for 5 minutes at 4 °C. 100 μl of supernatant was collected and mixed with 100 μl ATP detection solution. Luminance was measured by fluorescence microplate reader (Synergy4, BioTek, USA). The ATP level was normalized to the protein concentration of each sample.

### NAD+/NADH ratio measurement

The kidney tissues were homogenized on ice bath, and nicotinamide adenine dinucleotide (NAD) and reduced form of nicotinamide-adenine dinucleotid (NADH) were determined by using a commercial NAD^+^/NADH Assay Kit (BioAssay Systems, CA, USA). The results were expressed as NAD^+^/NADH ratio calculated from a standard curve generated from absorbance values at 565 nm.

### Quantitative real-time PCR (qPCR)

Total RNA was isolated from renal tissues by using Trizol (GIBCO, Life Technologies, USA) according to the manufacturer’s instructions. Total RNA was quantified by NanoDrop 2000spectrophotometer (Thermo Fisher Scientific Inc., USA). cDNA was synthesized by an iScript cDNA Synthesis Kit (Bio-Rad, CA, USA). The sequences of primers were listed in S1 Table qPCR analysis were performed on a CFX96 Real-Time PCR Detection System (Bio-Rad, CA, USA) with SYBR green supermix (SsoFast EvaGreen, Bio-Rad, USA). The change folders of mRNAs were calculated by delta-delta Ct method with β-actin as internal reference gene.

### Histological examination

The fixed renal tissues were embedded in paraffin and made to 5 μm sections. Renal sections were deparaffinized in xylene and rehydrated in graded ethanols, and then stained with hematoxylin eosin (HE) and periodic acid-schiff (PAS) staining. For immunohistochemical (IHC) staining, sections were blocked with 1% BSA, and incubated with diluted primary antibodies including rabbit anti-α-SMA (Abcam, USA), mouse anti-MCP-1 (Abcam, USA), rabbit anti-IL-6 (Abcam, USA), rabbit anti-CPT1 (Proteintech, USA), rabbit anti-FABP1 (Proteintech, USA), rabbit anti-PPARα (Proteintech, USA), rabbit anti-UCP2 (Abcam, USA), rabbit anti-ATP5A1 (ABClonal, USA), rabbit anti-Sirt1 (CST, USA), rabbit anti-p-AMPK (CST, USA) and rabbit anti-PGC1α (Proteintech, USA), then incubated with HRP-conjugated secondary antibody (DAKO, USA), and finally stained with DAB substrate and hematoxylin. The images of stained sections were acquired by microscope (Carl Zeiss, Germany), and quantitative analysis of positive staining areas (%) in images was done by using Image J software (NIH, USA).Five images of non-overlapping fields were captured from each section per animal, and the pathological score of renal injury including glomerulosclerosis, interstitial fibrosis and glomerular size were performed as previously described [20, 21].

### Statistical analysis

Descriptive statistics were presented as mean ± SD and analyzed by SPSS software (version 11.5, IBM Corp., USA). Comparison among groups was analyzed with one-way analysis of variance (ANOVA) and Turkey post-hoc test, and p < 0.05 was considered as significant difference.

## Results

### The effect of Lira on general and clinical parameters in HFD rats

As shown in Table 1, HFD rats had higher levels of body weight (BW), kidney weight (KW), BG, TC, TG, LDL-C and areas under the curve (AUC) of IPGTT (S1 Fig), and lower level of HDL-C compared to control. By contrast, Lira significantly reduced BW, KW, BG, AUC and blood lipid levels in HFD rats. In addition, HFD rats showed increased levels of BUN, CREA, CC and UACR compared to control, while Lira significantly reversed CREA, UACR and decline of CC in HFD rats. Meanwhile, Lira-treated HFD rats showed slight reduction of liver function parameters such as AST, ALT and CK compared to HFD rats.

**Table 1:** General and biochemical parameters of rats in different groups.

Parameters	Control	HFD	HFD + Lira
BW (g)	451.8±37.32	625.8±45.72**	512.6±49.31^#^
Kidney weight (g)	2.12±0.04	3.93±0.41**	2.71±0.32^#^
BG (mmol/L)	5.60±1.59	9.98±3.46**	7.45±2.69^#^
TC (mmol/L)	0.70±0.20	1.82±0.34**	1.12±0.14^##^
TG (mmol/L)	0.26±0.11	0.51±0.27**	0.33±0.14^#^
LDL-C (mmol/L)	0.18±0.06	0.98±0.33**	0.67±0.22^#^
HDL-C (mmol/L)	1.03±0.13	0.55±0.19**	0.79±0.24^#^
BUN (mmol/L)	4.24±0.86	5.88±1.92*	4.97±1.03
CREA (μmol/L)	35.18±10.95	76.66±21.43**	54.68±17.32^#^
UACR (mg/mmol)	2.34±0.73	8.80±3.15**	5.38±1.69^#^
CC (ml/min)	5.2±1.08	3.3±0.6**	4.5±0.82^#^
AST (IU/L)	120.11±18.43	180.53±19.58**	166.31±12.83
ALT (IU/L)	34.73±4.73	60.26±4.66**	49.71±7.44
CK (IU/L)	1094.12±86.61	1470.35±88.93**	1390.25±81.32

Note

* p<0.05

** p<0.01comparedwith control

# p<0.05

^# #^ p<0.01 compared with HFD.

### Lira reduced renal inflammation and fibrosis in HFD rats

**Fig 1:**
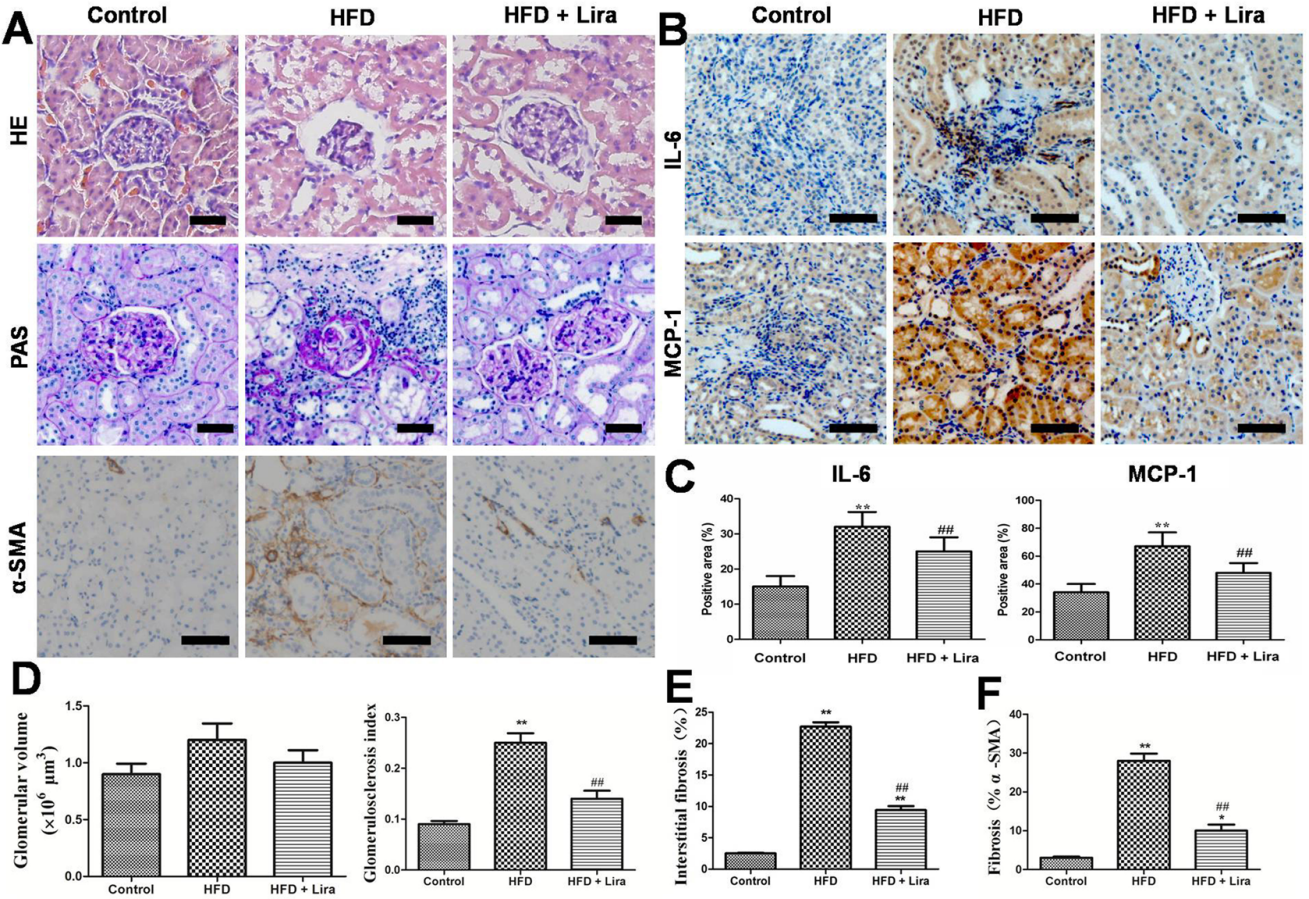
(A)Representative HE, PAS staining (scale bar = 50 μm) and IHC staining for α-SMA (scale bar = 100 μm) of kidney from control, HFD and HFD + Lira group. (B) IHC staining for IL-6 and MCP-1 in kidney of different groups (scale bar = 100 μm). (C)Quantification of IL-6 and MCP-1 protein detected by IHC staining. (D-E) Morphological analysis of glomerular size, glomerulosclerosis and interstitial fibrosis in renal sections. (F)Quantification of α-SMA detected by IHC staining(** p<0.01, compared with control;^#^ p<0.05,^# #^ p<0.01, compared with HFD).

HFD rats showed obvious renal lesions including tubular hypertrophy, basement membrane thickening, mesangial expansion and glomerulosclerosis compared to control rats (Fig 1A). In contrast, Lira markedly reduced renal pathological injuries including glomerular size, glomerulosclerosis and interstitial fibrosis in HFD rats (Fig 1D–1F). HFD rats had increased levels of monocyte chemotactic protein 1 (MCP-1), interleukin-6 (IL-6) and α-Smooth muscle actin (α-SMA), while Lira inhibited these pro-inflammatory and pro-fibrotic factors expression in the kidney of HFD rats (Fig 1B and 1C). In addition, Lira also reduced serum IL-6 and TGF-β1 levels in the HFD rats (S2 Fig).

### Lira ameliorated renal metabolic disorders in HFD rats

**Fig 2:**
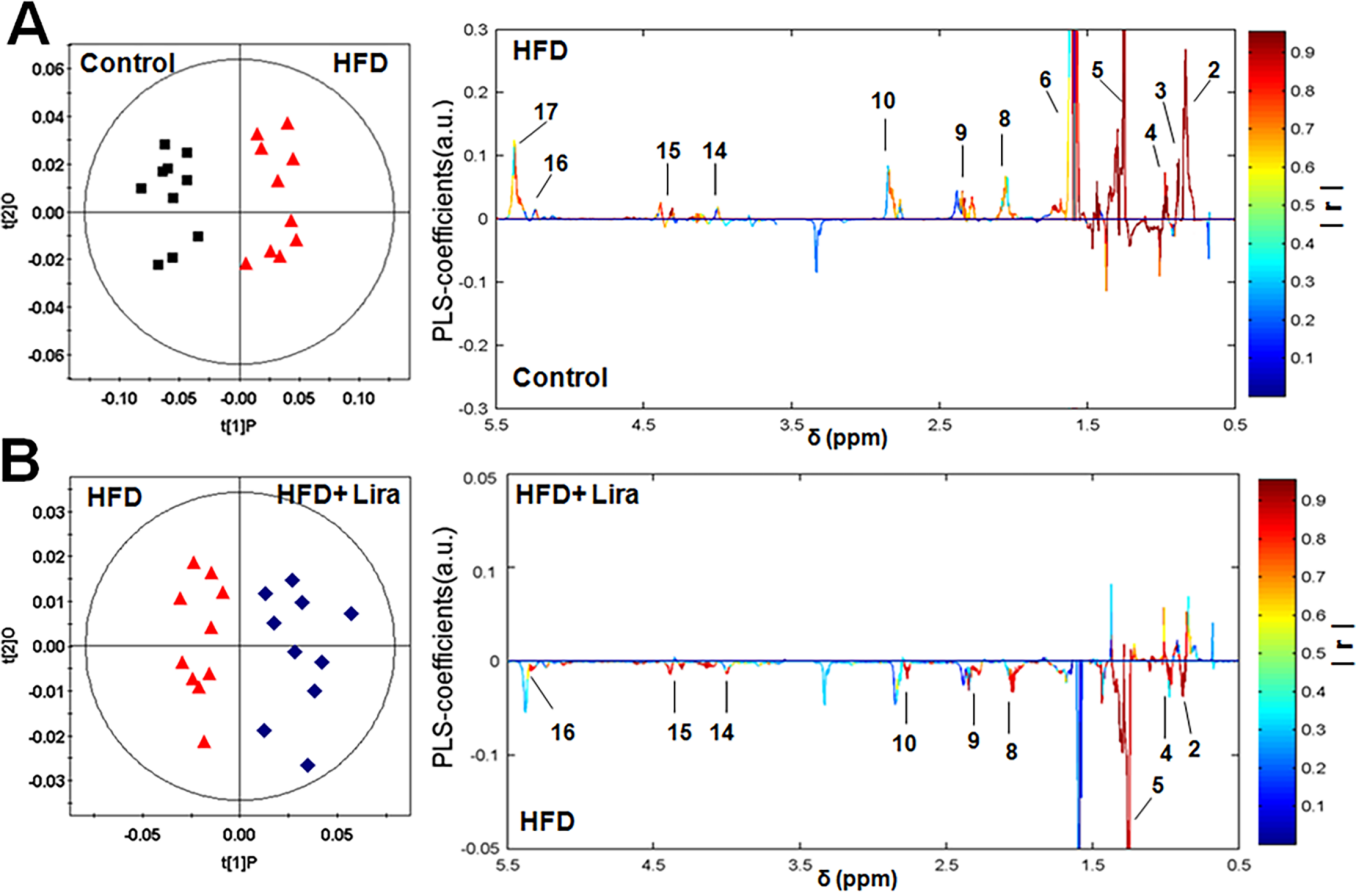
OPLS-DA loading plots derived from renal lipid extracts of ^1^H NMR spectra in (A) HFD group *vs* control group and (B) HFD+ Lira group *vs* HFD group. Note: 1. Total Cholesterol (C_18_H_3_), 2. Fatty acid residues (ω-CH_3_), 3. Total Cholesterol (C_26_H_3_, C_27_H_3_), 4. Free Cholesterol (C_19_H_3_), 5. Fatty acid residues ((CH_2_-)_n_), 6. Fatty acid residues (COCH_2_-CH_2_), 7. Fatty acid residues (-CH_2_ of ARA+EPA), 8. Fatty acid residues (CH_2_-CH = 9. Fatty acid residues (-CO-CH_2_), 10. Fatty acid residues (-CH = CH-CH_2_-CH = CH-), 11. Phosphatidylethanolamine (-CH_2_-NH_2_), 12. Sphingomyelin (-CH_2_-N-(CH_3_)_3_), 13, Phosphatidylcholine (-CH_2_-N-(CH_3_)_3_), 14. Total phospholipids (Glycerol (C_3_H_2_)), 15. Triglycerides (C_1_H and C_3_H of glycerol), 16. Triglycerides(C_2_H of glycerol), 17. Fatty acid residues (-CH = CH-).

**Fig 3:**
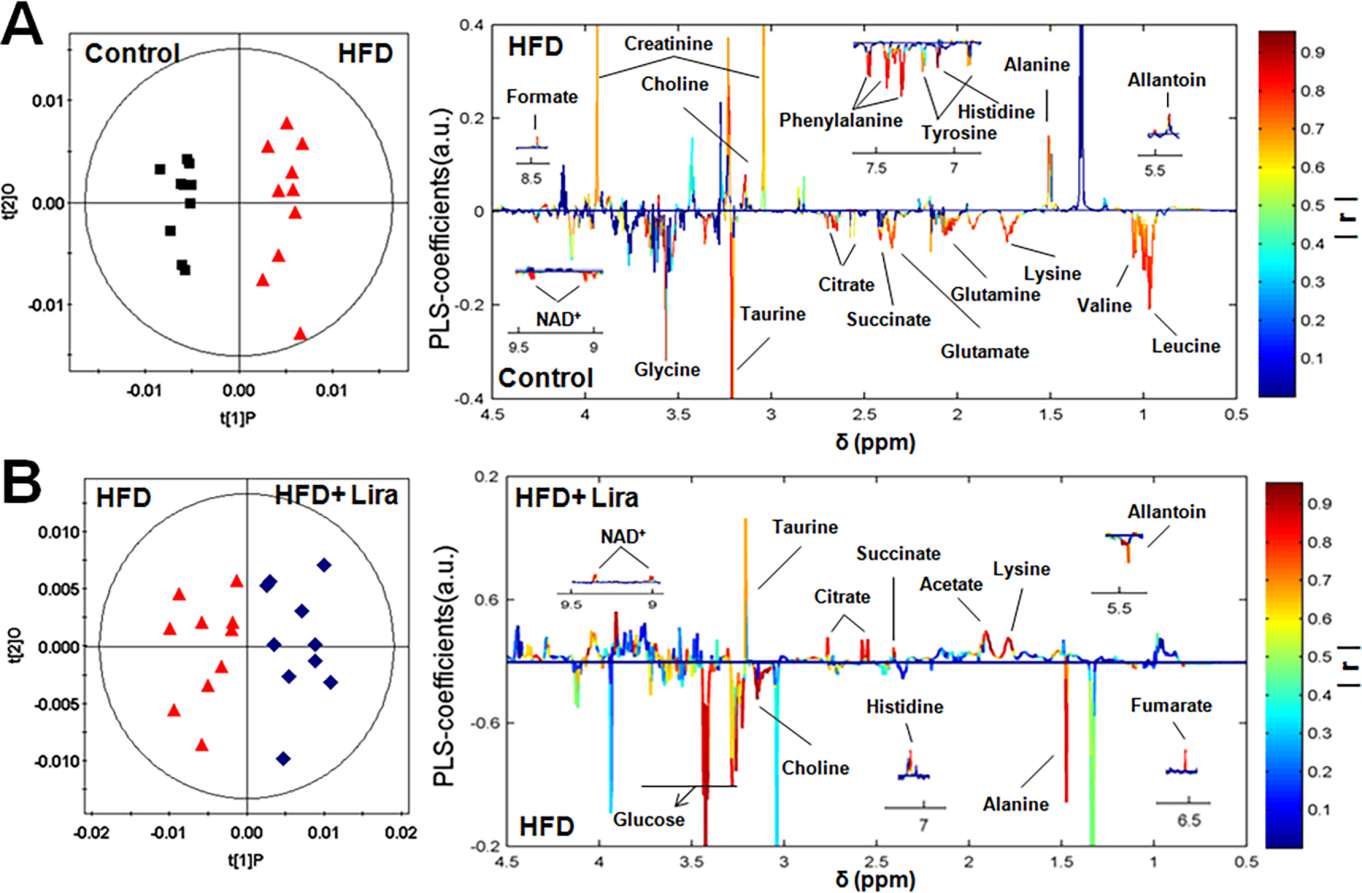
OPLS-DA loading plots derived from renal aqueous extracts of ^1^H NMR spectra in (A) HFD group *vs* control group and (B) HFD+ Lira group *vs* HFD group.The colored correlation coefficients indicated significant metabolites which were higher or lower in each group.

The representative ^1^H NMR spectra of lipid and aqueous extract from rat kidney were shown in S3 and S4 Figs, respectively. OPLS-DA score plots showed clear clustering among the three groups (Figs 2 and 3). In lipid extract, HFD rats showed higher level of fatty acid residues, cholesterol, phospholipids and triglycerides compared to controls, while Lira reduced these lipids levels in the kidney of HFD rats (Fig 2). In aqueous extract, HFD rats showed lower level of leucine/isoleucine, valine, lysine, glutamine, glutamate, succinate, citrate, taurine, glycine, tyrosine, histidine, phenylalanine and NAD^+^, and higher level of alanine, creatinine, choline, allantoin and formate compared to controls. In contrast, Lira reduced the level of alanine, choline, glucose and allantoin, and increased the level of lysine, acetate, succinate, citrate, taurine, fumarate, histidine and NAD^+^ in the kidney of HFD rats (Fig 3).

### Lira decreased renal lipids accumulation in HFD rats

**Fig 4:**
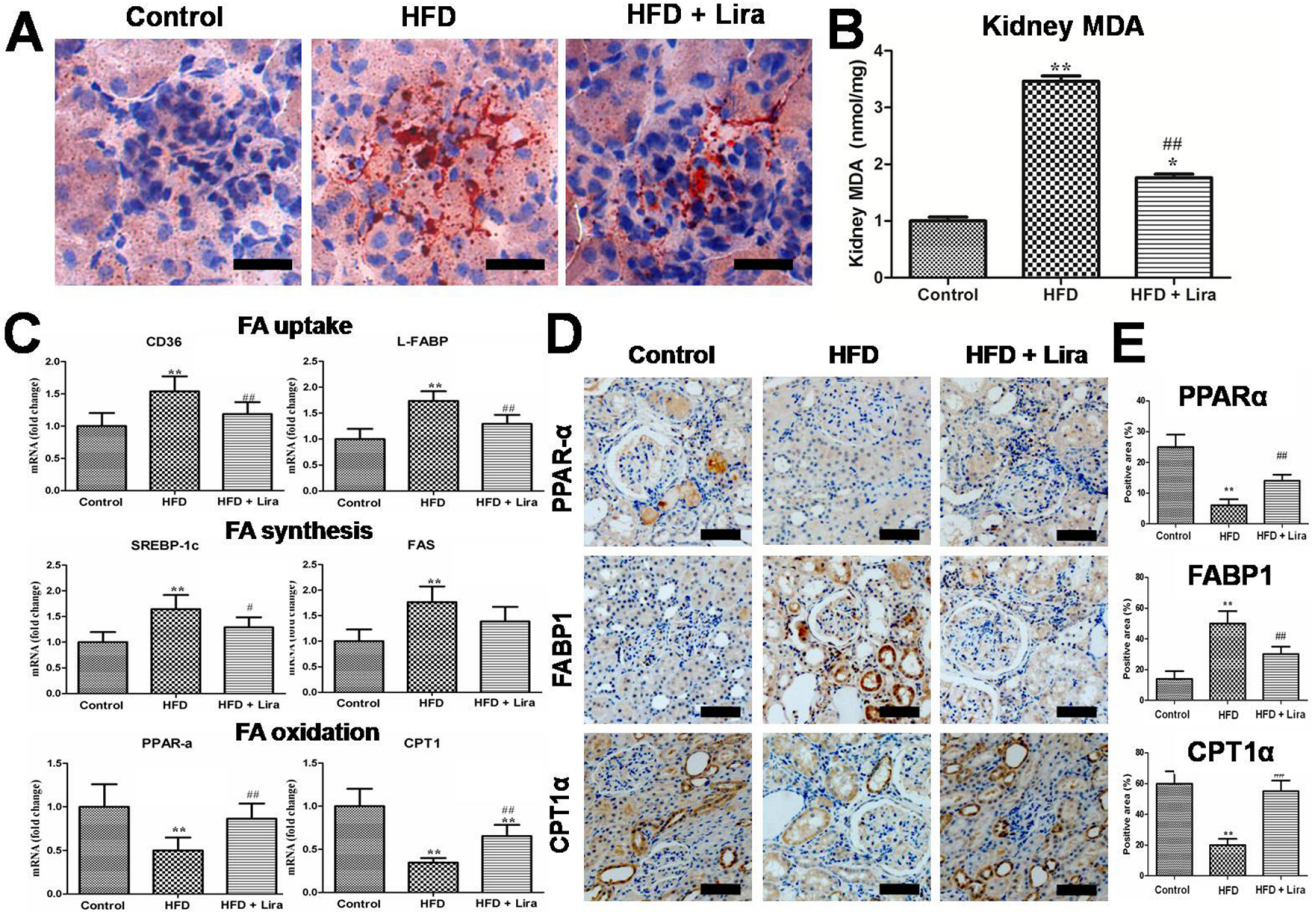
(A)Oil Red O (ORO) staining of kidney from control, HFD and HFD + Lira group (scale bar = 100 μm).(B) Quantification of kidney MDA level. (C) Real-time PCR analysis for CD36, L-FABP, SREBP-1c, FAS, PPAR-α and CPT1 mRNA. (D) IHC staining for renal PPAR-α, FABP1 and CPT1(scale bar = 100 μm). (E) Quantification of PPAR-α, FABP1 and CPT1proteindetected by IHC staining (* p<0.05, ** p<0.01, compared with control;^#^ p<0.05, ^# #^ p<0.01, compared with HFD).

Compared with controls, HFD rats showed increased lipid droplets formation (Fig 4A) and MDA (Fig 4B) in the kidney, while Lira reduced the lipid accumulation and lipid peroxidation in the obese kidney. Moreover, HFD rats had increased level of lipogenic genes including CD36, liver-type fatty acid binding protein (L-FABP), sterol regulatory element binding protein-1c (SREBP-1c) and fatty acid synthase (FAS), and reduced lipolytic genes including carnitine palmitoyl transferase 1(CPT-1) and peroxisome proliferators-activated receptors α (PPARα). By contrast, Lira significantly reduced these lipogenic genes and enhanced lipolytic genes expression in the kidney of HFD rats (Fig 4C). Lira also reduced fatty acid binding protein1 (FABP1) and increased PPAR-α and CPT1 protein level in kidney of HFD rats (Fig 4D and 4E).

### Lira restored renal mitochondrial function in HFD rats

**Fig 5:**
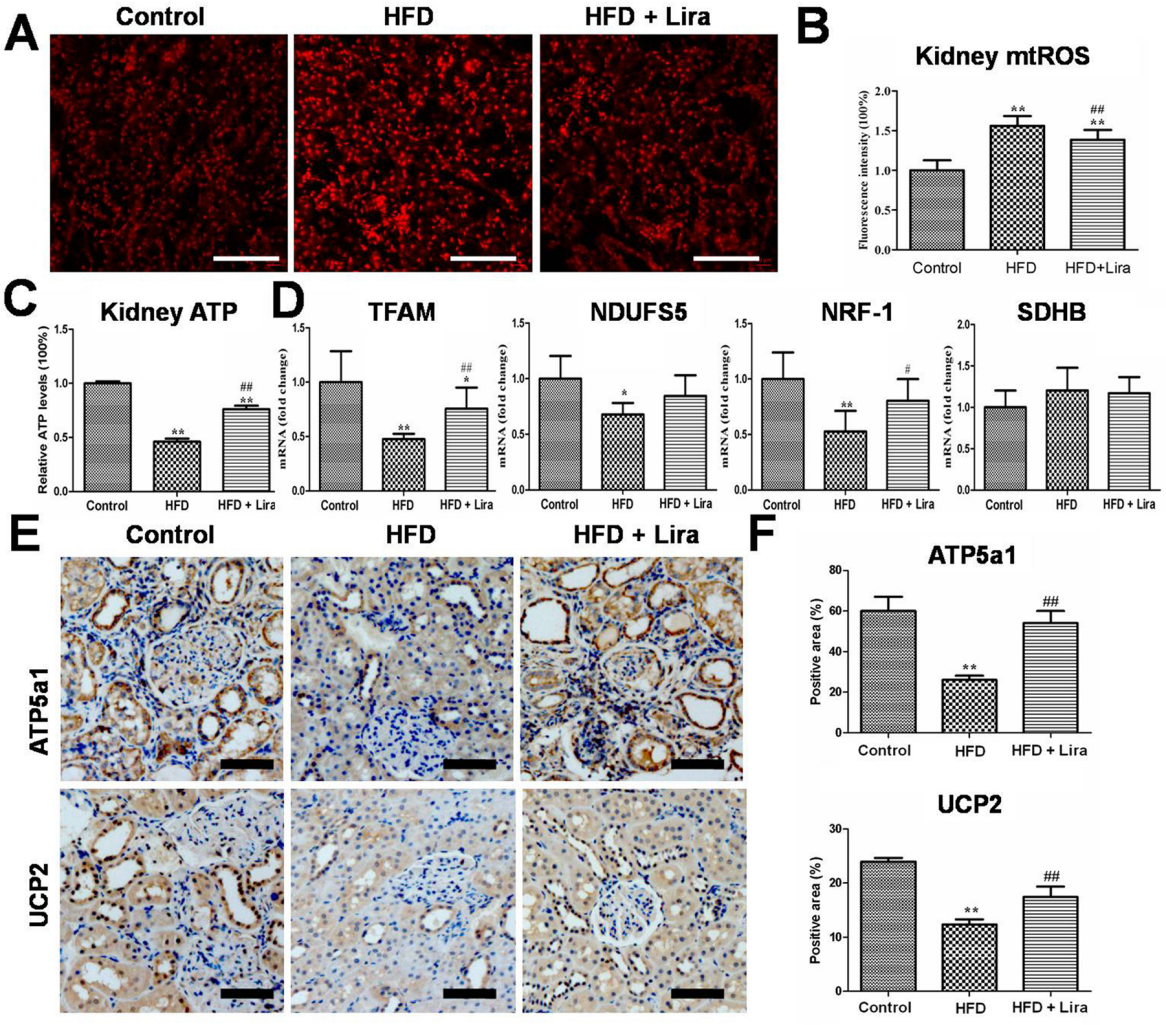
(A) Fluorescence staining of MitoSOX in kidney from control, HFD and HFD + Lira group (scale bar = 100μm). (B) Quantification of kidney mtROS detected by MitoSOX staining. (C) Renal tissue ATP levels in different groups. (D) Real-time PCR analysis for TFAM, NRF-1, NDUFS5 and SDHB mRNA. (E) IHC staining for renal UCP2 and ATP5a1(scale bar = 100μm). (F) Quantification of UCP2 and ATP5a1protein detected by IHC staining (* p<0.05, ** p<0.01 compared with control; ^#^ p<0.05,^# #^ p<0.01 compared with HFD).

HFD rats showed increased renal level of mitochondrial ROS (mtROS) (Fig 5A and 5B) and reduced level of ATP (Fig 5C), while Lira significantly inhibited mtROS and promoted ATP production in the kidney of HFD rats. In addition, Lira reversed the decline of mitochondria biogenesis genes including mitochondrial transcription factor A (TFAM), Nuclear respiratory factor-1(NRF-1), NADH dehydrogenase [ubiquinone] iron-sulfur protein 5 (NDUFS5) and succinate dehydrogenase [ubiquinone] iron-sulfur subunit(SDHB) expression in kidney of HFD rats (Fig 5D). IHC staining results showed that Lira increased ATP5a1 and mitochondrial uncoupling protein 2(UCP2) protein levels in the kidney of HFD rats (Fig 5E and 5F)

### Lira activated renal Sirt1/AMPK/PGC1α signals in HFD rats

**Fig 6:**
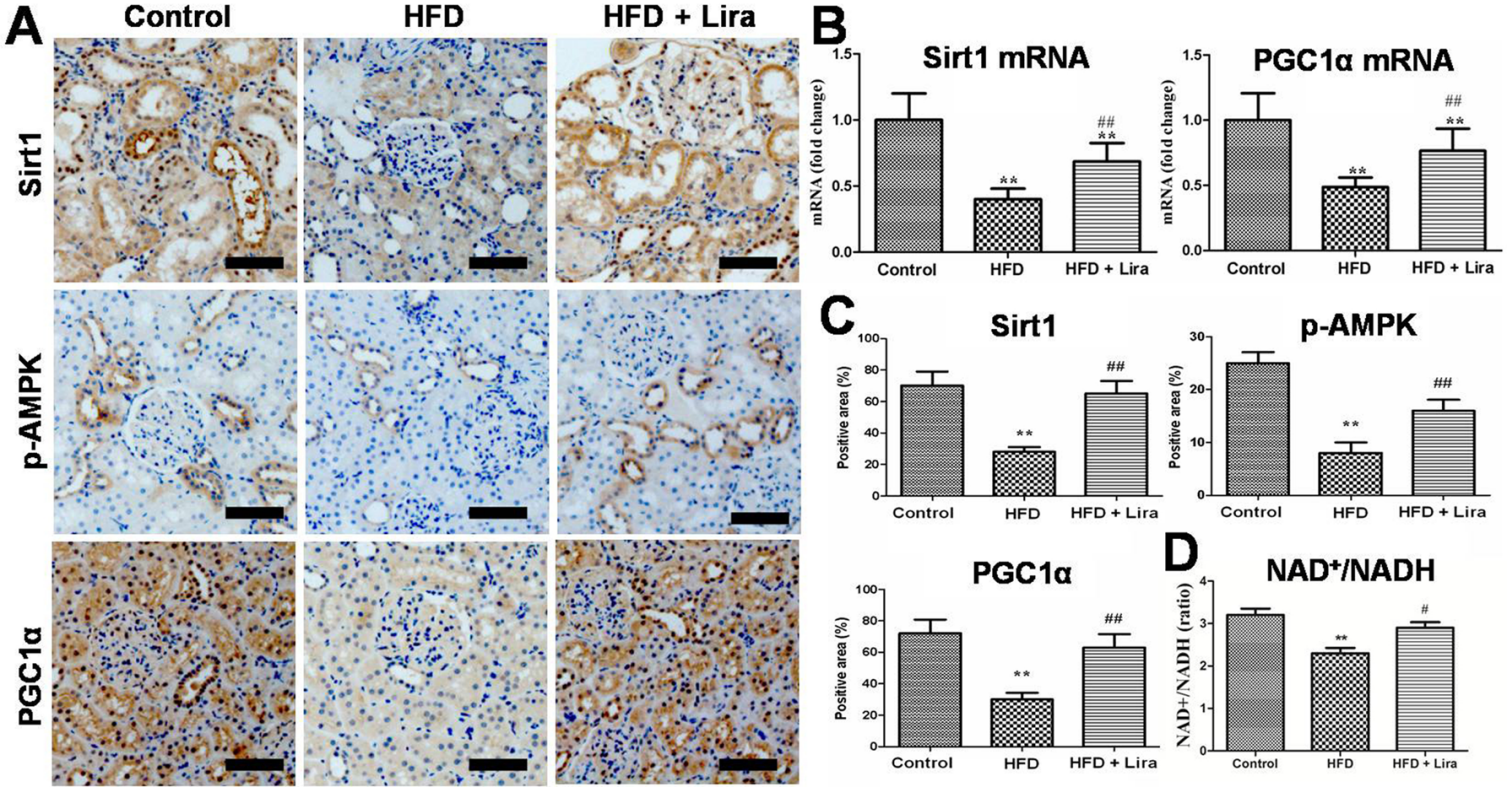
(A) IHC staining for PGC1α, p-AMPK and Sirt1of kidney from control, HFD and HFD + Lira group (scale bar = 100μm). (B) Real-time PCR analysis for Sirt1 and PGC1α mRNA. (C) Quantification of PGC1α, p-AMPK and Sirt1 protein detected by IHC staining. (D) Determination of renal NAD^+^/NADH ratio by commercial kit (** p<0.01 compared with control; ^# #^ p<0.01 compared with HFD).

As shown in Fig 6B, qPCR results showed that HFD rats had reduced mRNA levels of Sirtuin 1 (Sirt1) and peroxisome proliferator-activated receptor gamma coactivator 1-alpha (PGC1α) in kidney, while Lira significantly up-regulated these genes expression in the kidney of HFD rats. IHC staining results showed that HFD inhibited Sirt1, PGC1αand p-adenosine 5‘-monophosphate (AMP)-activated protein kinase (AMPK) expression in kidney, whereas Lira significantly increased these proteins expression in the kidney of HFD rats (Fig 6A and 6C). Furthermore, Lira also reversed the decline of renal NAD^+^/NADH ratio in HFD rats (Fig 6D).

## Discussion

High-fat diets (HFD) increased the prevalence of obesity and its associated metabolic syndrome, type 2 diabetes mellitus (T2DM) and cardiovascular diseases^2^. GLP-1R agonist had been proved to reduce food intake, body weight and blood glucose in obese rats[22]. Consisted with previous report, we observed that Lira reversed the general metabolic disorders including elevated body weight, hyperlipidemia and impaired glucose tolerance induced by HFD. Moreover, HFD rats showed clear kidney histological lesions, impaired renal function, and increased oxidative stress and inflammation, and these factors have been well recognized as key mediators for the development of CKD. The association between obesity and CKD has been already purposed, and HFD increased oxidative stress markers such as 8-OH-dG, and induced sustained inflammation and cell apoptosis in the kidney [23]. Previous study found that GLP-1R expressed on the renal glomeruli and endothelial cells, and GLP-1R therapy had beneficial effects on diabetic kidney diseases [24]. GLP-1R agonist Ex-4could attenuate renal oxidative stress, extracellular matrix (ECM) deposition and cytokines release in streptozotocin (STZ)-induced DN rats via direct effects on the GLP-1R in kidney tissue[25]. In this study, we observed that Lira significantly improved renal function, and ameliorated renal histological injury, pro-inflammatory and pro-fibrotic factors expression in HFD rats. These results again demonstrated that GLP-1R agonist Lira had renal protective role in obese rats.

The anti-inflammatory and antioxidant effects of GLP-1R agonist on kidney injury are well-known[11, 24, 25], but its direct effect on renal metabolism in obesity has rarely reported. To further explore the metabolic mechanism of Lira for kidney protection, the renal metabolic profile of HFD rats was performed. Our results showed that many metabolites were affected by HFD, and multiple of them were related to the disturbed glucose / protein metabolism because of the obesity and pre-diabetes status in HFD rats. For instance, alanine that elevated in HFD rats was an important intermediate in glucose metabolism, which could be converted into pyruvate and thus enhanced gluconeogenesis via glucose-alanine cycle [26]. The decline of amino acids such as leucine, valine, phenylalanine and tyrosine reflected the imbalance of protein catabolism and anabolism in HFD rats, which might be due to insulin resistance and activation of mTOR pathway [27].

Moreover, we found that some metabolites had been involved in kidney diseases as previous reports. For example, allantoin was an end product of purine metabolism from uric acid (UA). High level of UA was an independent risk factor for kidney and heart diseases, which caused renal injury via inducing oxidative stress and tubular epithelial mesenchymal transition (EMT)[28]. The increased choline in HFD rats suggested the gut microbiota disorders because dietary choline was mainly degraded by gut microbiota. Gut microbiota dysbiosis contributed to the development of obesity, insulin resistance and T2DM, and it could also induce intestinal barrier dysfunction and systemic inflammation in CKD patients [29]. Meanwhile, acetate was a metabolic product of gut microbiota, which had shown anti-inflammatory effects in inflammatory bowel disease [30], thus the increase of acetate suggested that Lira might improve gut microbiota function in HFD. The decline of glycine and taurine reflected the impaired antioxidant mechanism in HFD rats because they were well-known antioxidant substrates, which could prevent renal injury via antioxidant, cellular osmo regulation and antiapoptotic effects [31, 32].

Interestedly, we found that the renal protective role of Lira also dependent on other mechanism beyond the classic anti oxidant and anti-inflammatory mechanism. Briefly, lira could directly prevent lipid contents such as fatty acid residues, cholesterol, phospholipids and triglycerides deposition in the kidney of HFD rats—. Meanwhile, Lira could significantly reverse the decline of succinate, citrate, taurine, fumarate and NAD^+^ in the kidney of HFD rats. It is well-documented that succinate, citrate and fumarate were converted in TCA cycle of mitochondria [33], and thus their reduction reflected the disturbed energy metabolism and mitochondria dysfunction in kidney of HFD rats. In addition, NAD^+^ is an important substrate required for oxidative phosphorylation and mitochondrial respiration, and reduced NAD^+^ has been linked to oxidative stress, energy imbalance and down-regulation of sirtuins in obesity, metabolic syndrome and diabetes[34]. Therefore, our results suggested that modulation of lipid and energy metabolism were also involved in the renal protection of Lira on HFD rats.

Previous studies had found abnormal lipid deposition in the kidney of metabolic diseases such as obesity and diabetes, and suggested that lipotoxicity contributes to the development of kidney injury such as glomerulopathy, tubule interstitial inflammation, tubular cell apoptosis and renal fibrosis [35]. In this study, we found that Lira could markedly reduced renal pathological injuries including glomerulosclerosis and interstitial fibrosis. Fatty acids (FAs) are key mediators of lipotoxicity, and their levels are dramatically elevated in obese patients [36]. The abnormally accumulated FAs lead to kidney injury through complex mechanism including ROS, ER stress, mitochondria dysfunction and activation of pro-inflammatory and pro-fibrotic pathways[37]. In this study, we found that Lira could directly regulate FA metabolism in obese kidney. SREBP-1 is a master regulators of lipid metabolism, which promotes lipogenesis by inducing target genes involved in the FA transport (CD36, FABP1) and FA biosynthesis (ACC and SCD-1)[37, 38]. In contrast, PPARα improves lipolysis via increasing genes related to FA oxidation such as CPT-1[39]. We observed that Lira inhibited SREBP-1c, FAS, CD36 and FABP1, and increased CPT-1 and PPARα level in the kidney of HFD rats. Thus, our results suggested that Lira alleviated kidney injury in obese rats at least partly by directly preventing renal lipogenesis and promoting FA oxidation.

The kidney is a high energy demand organ that can use various substrates as fuels to generate ATP. In renal tubule cells, mitochondrial oxidation of FFA is a major source for ATP production due to their little glycolytic capacity[36]. Obesity exhibited energy depletion and mitochondrial dysfunction associated with declined key mitochondria regulator proteins including PGC1α and AMPK[3, 40, 41]. Previous reports indicated that renal accumulated lipids caused mitochondria stress including increased mtROS, depolarized mitochondrial membrane potential and impaired ATP generation in kidney cells [42, 43]. In this study, we observed that Lira partly decreased renal mitochondria injury induced by HFD, as indicated by reduced mtROS and increased level of TCA metabolites, ATP, NAD^+^ and mitochondria biogenesis genes such as TFAM, NRF-1, NDUFS5 and SDHB, and increased ATP5a and UCP2 protein in kidney of HFD rats.Sirt1 is a NAD^+^ dependent deacetylase, which plays a key role in regulating mitochondria oxidative metabolism and energy homeostasis [34]. Sirt1 had been reported to regulate energy metabolism and mitochondria biogenesis through a complex network involving AMPK and PGC1α[44]. GLP-1R agonist exenatide had shown to activate Sirt1/AMPK pathways and thus reduced lipid deposition and inflammation in the liver of HFD mice [45]. We also observed that Lira could reverse the decline of NAD^+^ and Sirt1/p-AMPK/PGC1α in the kidney of HFD rats. Therefore, our results suggested that Lira prevented kidney injury at least partly by restoring mitochondrial function and activating Sirt1/AMPK/PGC1α pathways in HFD rats.

## Conclusion

In summary, HFD-induced obese rats showed impaired renal function and elevated renal inflammation, oxidative stress and fibrosis, whereas Lira significantly ameliorated these adverse effects in HFD rats. Metabolomic results indicated that Lira could directly reduce renal lipid and energy metabolic disorders in HFD rats. Furthermore, we revealed that Lira inhibited renal lipids deposition by coordinating lipogenic and lipolytic signals, and partly restored renal mitochondria function via Sirt1/AMPK/PGC1α pathways in HFD rats. Our findings suggested that GLP-1R agonist is a promising therapy for obesity-associated CKD.

## Supporting information

S1 Fig (A) The IPGTT test in control, HFD and HFD + Lira rats. (B) The AUC of each group driven from IPGTT test (** p<0.01 compared with control; # p<0.05 compared with HFD).
(TIF)

S2 Fig Evaluation of (A) IL-6 and (B) TGF-β in the serum of rats by ELISA method (** p<0.01 compared with control; # p<0.05 compared with HFD; ## p<0.001 compared with HFD).
(TIF)

S3 Fig Representative 600 MHz ^1^H NMR spectra of kidney lipid extract from normal control rat, the keys for identified metabolites were listed. (TIF)

S4 Fig Representative 600 MHz ^1^H NMR spectra of kidneyaqueous extract from normal control rat, the identified metabolites were marked. (TIF)

S1 Table Primers sequences for real-time PCR in this study. (DOCX)

S1 File Supporting methods. (DOCX)
