# Supplementary figures and images for: GLP-1 receptor agonist ameliorates obesity-induced chronic kidney injury via restoring renal metabolism homeostasis
Source: PLoS One. 2018 Mar 28;13(3):e0193473. doi: 10.1371/journal.pone.0193473 (PMC5873987; doi:10.1371/journal.pone.0193473)

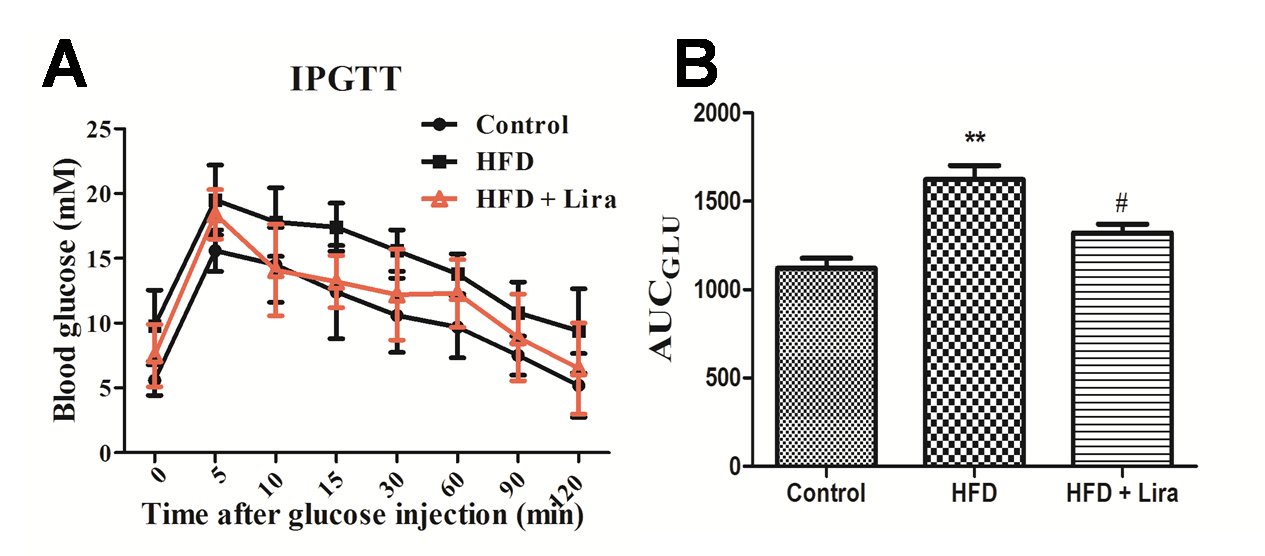

Supplement: S1 Fig — (A) The IPGTT test in control, HFD and HFD + Lira rats. (B) The AUC of each group driven from IPGTT test (** p<0.01 compared with control; # p<0.05 compared with HFD). (TIF) [file pone.0193473.s001.tif]

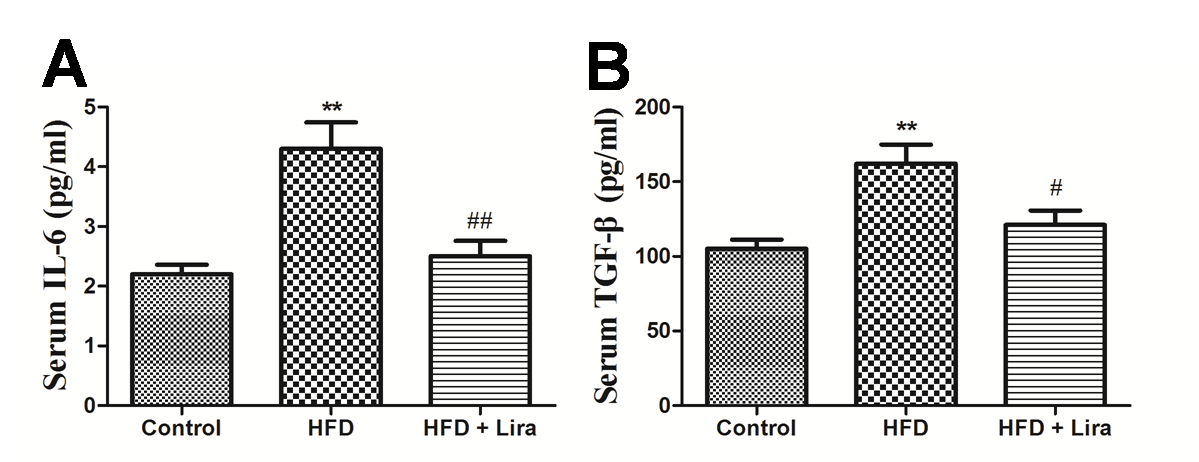

Supplement: S2 Fig — Evaluation of (A) IL-6 and (B) TGF-β in the serum of rats by ELISA method (** p<0.01 compared with control; # p<0.05 compared with HFD; ## p<0.001 compared with HFD). (TIF) [file pone.0193473.s002.tif]

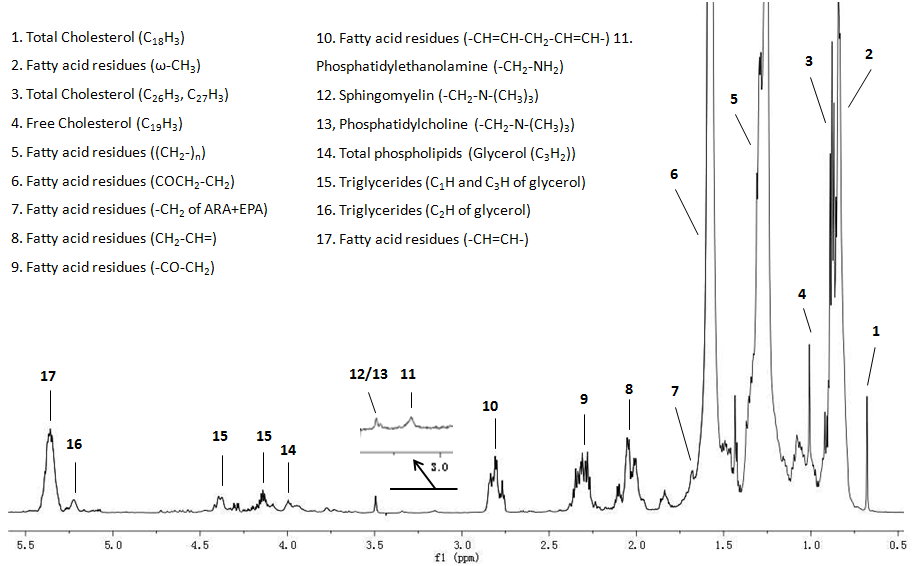

Supplement: S3 Fig — (TIF) [file pone.0193473.s003.tif]

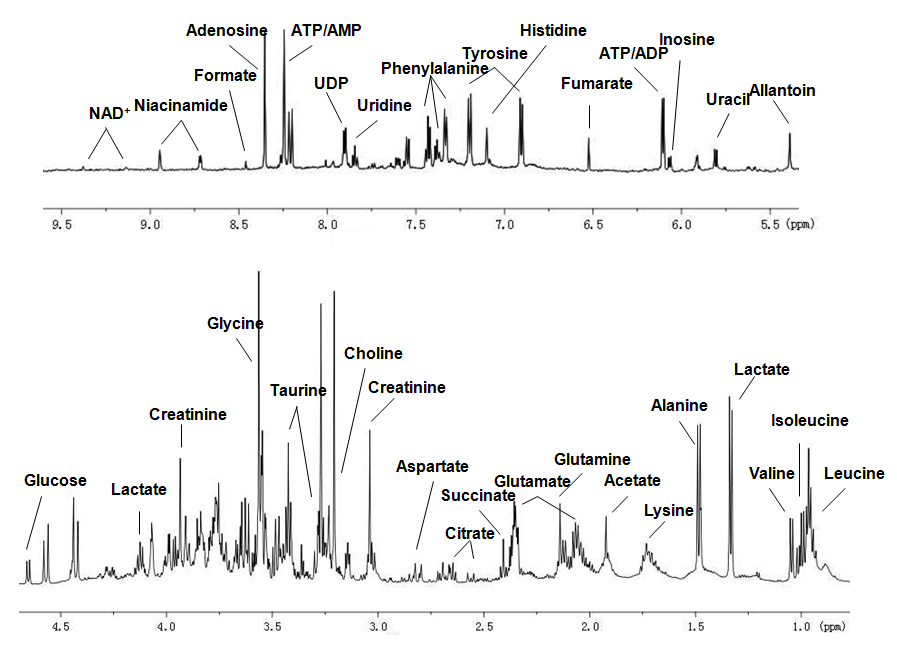

Supplement: S4 Fig — (TIF) [file pone.0193473.s004.tif]
