# Supplementary material for: GLP-1 receptor agonist ameliorates obesity-induced chronic kidney injury via restoring renal metabolism homeostasis
Source: PLoS One. 2018 Mar 28;13(3):e0193473. doi: 10.1371/journal.pone.0193473 (PMC5873987; doi:10.1371/journal.pone.0193473)
Supplement: S1 Table — (DOCX) [file pone.0193473.s005.docx]

**S1 Table**. Primers sequences for real-time PCR in this study

| Gene | Forward (5’-3’) | Reverse (5’-3’) |
| --- | --- | --- |
| CD36 | TGGATGAGCCTACATTATGCACT | GCACACCACCGTTTCTTCAA |
| L-FABP | GAGGGAGATCTATTGCCACCATGAGTTTC | GAAATGGGTACCTGTTTAAATTCTCTTGC |
| SREBP-1c | GGAGCCACAATGAAGACCGC | GTGGATGGGCAGTTTGTCTGT |
| FAS | GAGAGCCTGCCACCTATGAC | ACGGTTCCTCTCAACACCTG |
| PPAR-α | ATTCGGCTAAAGCTGGCGTA | TGCATTGTGTGACATCCCGA |
| CPT1 | CCACGAAGCCCTCAAACAGA | GGTCCGACTGATCTTTGCGA |
| ATP5a1 | GCCCAAACCAGGGCTATGAA | TCAGAACCGAACTGGGCAAA |
| TFAM | TTCCAGGGGGCTAAGGATGA | CACACTGCGACGGATGAGAT |
| NRF-1 | AGCCGTTGGAGCACTTACTG | TCACGGCTTTGCTGATGGT |
| NDUFS5 | GCCGGACTACACGCTATGAC | CCCTCCACGATAGCGTCAAC |
| SDHb | CGACCTACAAGGAGAAGCGG | TTGAAGGGACTCACGCCAGA |
| Sirt1 | TCATTCCTGTGAAAGTGATGACGA | CTGCCCTAGTGTCATATCATCCAA |
| PGC1α | CCAGTCTACGGCTGTTTGGT | TGGAAGAACAGATGTGCCCC |
| β-actin | GGAGATTACTGCCCTGGCTCCTA | GACTCATCGTACTCCTGCTTGCTG |
