# Supplementary material for: GLP-1 receptor agonist ameliorates obesity-induced chronic kidney injury via restoring renal metabolism homeostasis
Source: PLoS One. 2018 Mar 28;13(3):e0193473. doi: 10.1371/journal.pone.0193473 (PMC5873987; doi:10.1371/journal.pone.0193473)
Supplement: S1 File — (DOCX) [file pone.0193473.s006.docx]

Supplementary methods

Intraperitoneal glucose tolerance test (IPGTT)

After fasting overnight, the rats were given glucose (0.5 g/kg) by intraperitoneal injection. The blood glucose (BG) concentration was measured at 0, 5, 10, 15, 30, 60, 90 and 120 minutes after glucose injection.

ELISA assay

The levels of IL-6 and TGF-β in the serum of each group were detected by ELISA (NeoBioscience Technology, Guangzhou, China). All operations were performed in strict accordance with the instructions of the kit. Each sample was tested three times with two technique repetitions performed each time.
